# The Effects of Plyometric Training on Selected Neuromuscular Outcomes in Trained-to-Highly Trained Male Boulder Climbers: A Preliminary Exploratory Trial

**DOI:** 10.3390/sports14080313

**Published:** 2026-07-23

**Authors:** Guillermo Cortés-Roco, Verónica Low-Barría, Rodrigo Yáñez-Sepúlveda, Jorge Pérez-Contreras, Yeny Concha-Cisternas, Juan Hurtado-Almonacid, Exal Garcia-Carrillo

**Affiliations:** 1 Facultad de Ciencias de la Vida, Universidad Viña del Mar, Viña del Mar 2520000, Chile; kine.verolow@gmail.com; 2 Faculty of Education and Social Sciences, Universidad Andrés Bello, Viña del Mar 2520000, Chile; rodrigo.yanez.s@unab.cl; 3 School of Medicine, Universidad Espíritu Santo, Samborondón 0901-952, Ecuador; 4 Escuela de Ciencias del Deporte, Facultad de Salud, Universidad Santo Tomás, Santiago 8370003, Chile; joperezc@gmail.com; 5 Vicerrectoría de Investigación e Innovación, Universidad Arturo Prat, Iquique 1100000, Chile; yenyconchaci@santotomas.cl; 6 Escuela de Pedagogía en Educación Física, Facultad de Educación, Universidad Autónoma de Chile, Talca 3460000, Chile; 7 Escuela de Educación Física, Pontificia Universidad Católica de Valparaíso, Valparaíso 2340000, Chile; juan.hurtado@pucv.cl; 8 Department of Physical Activity Sciences, Faculty of Education Sciences, Universidad Católica del Maule, Talca 3480112, Chile; exal.garcia@gmail.com; 9 Department of Physical Activity Sciences, Universidad de Los Lagos, Osorno 5290000, Chile

**Keywords:** plyometric exercise, mountaineering, upper extremity, isometric contraction, neuromuscular performance, human physical conditioning, movement, muscle strength

## Abstract

Plyometric training is proposed as a method for improving neuromuscular performance in climbing; however, its specific effects on upper-body explosive power and rate of force development (RFD) in male boulderers, ranging from trained to highly trained, remain unclear. This preliminary exploratory trial examined the effects of a 10-week plyometric training program on selected laboratory-based neuromuscular performance outcomes. Eighteen male climbers were assigned to: intervention group (n = 9, 29.7 ± 4.5 yr) or a control group (n = 9, 31.3 ± 5.6 yr). Finger flexor strength and RFD (20-mm grip, 0–200 ms, 20–80%), isometric pull-up strength and RFD (load cell), upper-body power (plyometric push-up, Power Slap), and lower-body power (CMJ) were assessed. The intervention comprised two plyometric sessions/week for 10 weeks. Significant differences were observed in pull-ups (Δ difference = +3.89 repetitions; 95% CI: 0.30, 7.48; *p* = 0.036; η^2^p = 0.248), push-up power (Δ difference = +174.25 W; 95% CI: 5.05, 343.46; *p* = 0.044; η^2^p = 0.230), isometric pull-up RFD 0–200 ms (Δ difference = +107.85 kg/s; 95% CI: 27.54, 188.16; *p* = 0.012; η^2^p = 0.336), and the 20–80% range (Δ difference = +261.78 kg/s; 95% CI: 23.09, 500.47; *p* = 0.034; η^2^p = 0.253). No clear between-group differences were observed for finger flexor strength, finger RFD, maximum isometric pull-up strength, Power Slap, or CMJ. Given the exploratory design, small sample size, and low reliability of RFD-derived variables, these findings should be interpreted cautiously as preliminary evidence of movement-specific neuromuscular performance improvements.

## 1. Introduction

Bouldering is characterized by short, technically demanding movement sequences that require high levels of relative strength, body tension, grip strength, and rapid force application [1,2]. Unlike endurance-oriented climbing disciplines, many bouldering actions involve dynamic or reactive movements in which force must be generated within very brief time intervals [1,3]. Systematic reviews and methodological studies have shown that climbing performance is associated with a combination of anthropometric, strength, endurance, and neuromuscular factors, including finger flexor strength, upper-body pulling capacity, and climbing-specific force–time characteristics [4,5,6,7]. Consequently, laboratory measurements of explosive force production and RFD can provide useful information about certain neuromuscular qualities relevant to bouldering, although they should not be interpreted as direct measures of climbing performance.

RFD is defined as the slope of the force–time curve during the initial phase of contraction and is commonly used to describe the ability to generate force rapidly [8]. Differences in climbers’ performance may depend not only on the magnitude of maximum force but also on how force is expressed during brief, high-demand actions [1,9,10]. Maximum upper-body strength and RFD vary according to performance levels and are relevant to the expression of climbing-specific force [10]. The dynamic movements of climbing also require intersegmental coordination and, in some actions, lower-limb propulsion and control of the center of mass [11,12,13,14,15]. However, the relationship between laboratory-measured RFD and actual climbing performance remains indirect, particularly when ecological outcomes, such as problems completed, attempts required, route-specific performance, or competition performance, are not assessed.

Although bouldering has often been associated primarily with upper-body and finger strength, modern dynamic problems also require lower-body propulsion, coordinated body movement, and effective control of the center of mass [11,12,13,14,15]. Explosive movements of the lower limbs can contribute to certain dynamic climbing movements, particularly those involving large shifts in the center of mass [11,12,13,14], while upper-body pulling ability remains important for force generation and body position control [9,15]. For this reason, a program that includes plyometric exercises for both the upper and lower body may be justified from a multisegmented perspective. However, the expected transfer must remain specific to the characteristics of the stimulus and should be evaluated using clearly defined laboratory outcomes rather than assuming that it represents a direct improvement in climbing performance.

Plyometric training is widely used to develop explosive force production through stretch-shortening cycle actions and rapid transitions between eccentric and concentric muscle contractions [16,17,18,19,20]. Previous studies have reported that plyometric training can improve jumping performance and explosive power in various sports contexts [16,17], while for upper-body power tests, ballistic push-up protocols are used to assess the ability to apply force in the upper limbs [21,22]. However, in rock climbing, the evidence regarding such interventions remains limited. Shih et al. [23] analyzed a combined plyometric and strength training intervention in climbers, and Stien et al. [24] evaluated different training volumes using a campus board for advanced and elite climbers. These studies provide relevant background, but the specific effects of a structured plyometric program on isometric pull-up RFD, finger RFD, and select upper- and lower-body laboratory measures in male boulder climbers ranging from trained to highly trained remain insufficiently described.

The objective of this preliminary exploratory study was to determine the effects of a 10-week plyometric training program on specific neuromuscular performance outcomes in male boulder climbers ranging from trained-to-highly trained. Based on the principle of training specificity and the predominantly pull-oriented nature of the intervention, we hypothesized that the program would be associated with greater improvements in isometric pull-up RFD and upper-body explosive power in the intervention group compared to the control group. Conversely, no clear between-groups differences were expected for finger flexor strength, finger RFD, or CMJ, given the absence of a direct finger-specific loading and the program’s limited lower limb specificity.

## 2. Materials and Methods

### 2.1. Design

A randomized, parallel-group, controlled trial with repeated measures (group × time) was conducted over ten weeks.

### 2.2. Sample

**Figure 1:**
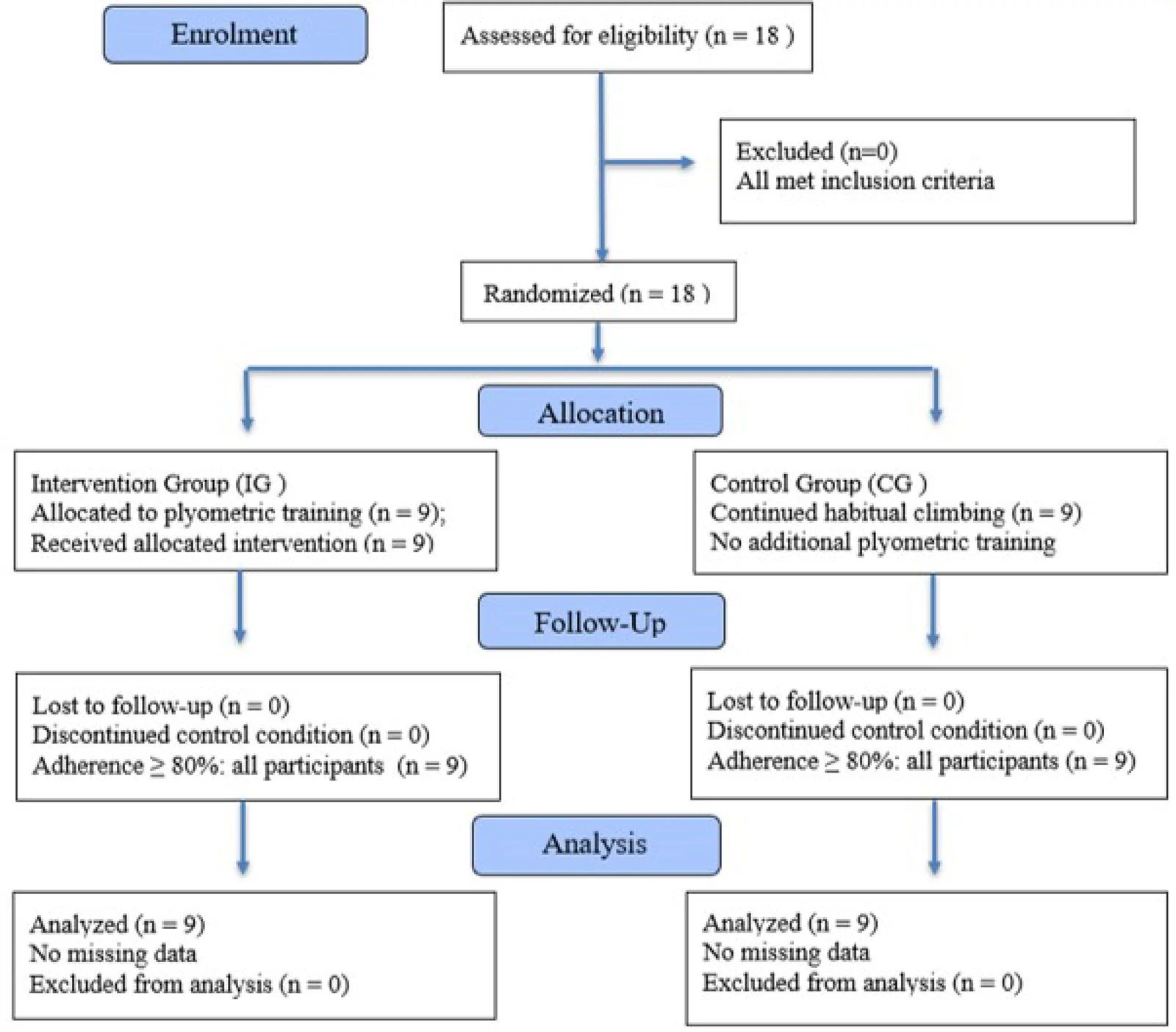
CONSORT-style flow diagram of participant enrolment, allocation, follow-up, and analysis. IG: intervention group; CG: control group. All randomized participants completed the study and post-intervention assessments. No missing outcome data or post-randomization exclusions were recorded.

Eighteen male climbers, with fitness levels ranging from trained to highly trained, were randomly assigned to an intervention group (IG, n = 9) or a control group (CG, n = 9) using a computer-generated random sequence. The allocation sequence was generated prior to group assignment and was not available to the external evaluator who conducted the assessments. The IG followed a plyometric training program twice a week for 10 weeks, while the CG continued their usual climbing training without additional plyometric training. All pre- and post-intervention assessments were conducted by an external evaluator who was unaware of the group assignments (single-blind design). Participants and intervention supervisors were necessarily aware of the group assignments. All participants had a minimum bouldering level of V5, defined as self-reported current maximum redpoint grade ≥ V5, at least two years of continuous experience in sport climbing, and a training frequency of three or more sessions per week. According to McKay et al. [25], the participants corresponded to levels 2–3 (trained-to-highly trained, regional/national level). According to the IRCRA climbing grade equivalence framework, V5 corresponds to the Advanced category (level 3), and the sample ranged from V5 to V9. No regular training with a campus board or hangboard was reported, and approximately 80% stated they had no prior experience with plyometric training. Figure 1 shows a CONSORT-style flow diagram of the participants; all 18 randomly assigned participants completed the study (100% retention). No a priori sample size calculation was performed; the sample size was determined based on the practical availability of eligible participants at a single climbing club. Given the exploratory nature of this study and the absence of a priori power analysis, all findings should be interpreted as hypothesis-generating rather than confirmatory.

### 2.3. Inclusion Criteria

Male climbers aged between 20 and 40 years were considered eligible if they had a minimum bouldering performance level of ≥V5, at least two years of continuous sport climbing experience, and a regular training frequency of three or more sessions per week over the previous six months.

### 2.4. Exclusion Criteria

The absence of active musculoskeletal injuries at the time of the initial assessment was required, with the aim of ensuring comparable baseline conditions and reducing the risk of adverse events during the plyometric program. Participants with medical conditions or ailments contraindicating reactive or ballistic training were excluded, as were those who sustained injuries during the intervention period that prevented continuation of the protocol. In addition, subjects with an adherence rate of less than 80% of the scheduled sessions were excluded.

Baseline characteristics included age, body mass, and body composition. Body composition was assessed by electrical bioimpedance using an InBody 270S^®^ multi-frequency analyzer (InBody Co., Seoul, Republic of Korea). Measurements were taken under standardized conditions, including prior fasting, abstention from vigorous exercise in the preceding 24 h, and control of hydration (Table 1).

### 2.5. Instruments

#### 2.5.1. Finger Strength on a 20 mm Hold

A Tindeq Progressor^®^ (Tindeq, Trondheim, Norway) load cell attached to a standard 20 mm hold was used to assess maximum finger strength and RFD. This device has demonstrated adequate validity and reliability for isometric strength measurements in climbing [26]. The protocol consisted of maximum isometric contractions with a standardized posture, recording the complete force–time curve for subsequent analysis. Signals were acquired at 80 Hz and filtered using a fourth-order Butterworth filter (low-pass, 10 Hz). The onset of contraction was defined as the point at which the force exceeded 5% above baseline. From the processed signal, Fmax, RFD 0–200 ms, and RFD 20–80% were calculated, including their normalization relative to Fmax and body mass. The sampling frequency of 80 Hz reflects the technical specifications of the Tindeq Progressor^®^ device; to mitigate the associated temporal resolution limitations, the analysis was restricted to the intervals of 0–200 ms and 20–80% of Fmax (16 samples at 80 Hz), and the initial phase windows (0–50 ms) were not analyzed.

#### 2.5.2. Pull-Up Repetitions and Maximum Isometric Strength in Pull-Ups

Pull-up repetition performance and maximum isometric strength in pull-ups were assessed using standardized procedures. For the pull-up repetition test, participants used a pronated grip, started from a full hanging position with elbows extended, and performed repetitions until voluntary failure. A valid repetition required the chin to pass above the bar without kipping, excessive trunk swing, or lower-limb assistance. For the isometric pull-up test, the Tindeq Progressor^®^ load cell was attached to the bar, and participants performed a maximal pull from a standardized pronated hanging position against a fixed resistance, with the elbows slightly flexed and the trunk aligned. Constant verbal encouragement was provided. The force–time curve was recorded following methodological recommendations for the analysis of force–time variables [8]. Signal processing, the definition of the onset of force, and the calculation of Fmax, RFD from 0 to 200 ms, and RFD from 20 to 80% of Fmax followed the same criteria described for finger strength, including normalization where appropriate.

#### 2.5.3. Lower-Body Power

Lower limb power was assessed using the Countermovement Jump (CMJ) test on a Chronojump^®^ contact platform (Boscosystem, Barcelona, Spain). Jump height (cm) was recorded based on flight time, and estimated power (W) was calculated. Participants performed the test with their hands on their hips to minimize the contribution of arm swing.

#### 2.5.4. Specific Power of the Upper Limbs

Upper limb power was assessed using the plyometric push-up test performed on a Chronojump^®^ contact platform (Boscosystem, Barcelona, Spain), recording flight time to estimate variables such as height (cm) and power (W), as suggested by previous studies [21,22]. Push-up power was estimated from flight time using total body mass, following Wang et al. [21] and Dhahbi et al. [22]. In the starting position, each athlete was asked to place both hands on the contact platform in a prone plank position, shoulder-width apart, with elbows extended, the trunk aligned, and feet flat on the ground. From this position, the athlete performed controlled elbow flexion to approximately 90°, followed immediately by explosive arm extension, lifting their hands off the platform. During the aerial phase, participants were instructed to keep the body aligned and avoid hip flexion. Flight time was recorded from the loss of contact with the platform until contact was regained. Three attempts were made, with 1–2 min of recovery between each. The best value was recorded for analysis. An attempt was considered valid when there was clear lift-off of both hands and no compensatory movements (hip flexion or arm asynchrony).

Upper-body specific power was assessed using the Power Slap test on a campus board, recording the distance achieved in centimeters, a procedure described previously [1,27]. The athletes started from a static hanging position on a standardized bar, with both hands using a pronated grip, shoulder-width apart, elbows extended, and without support from the lower limbs. From this position, they were asked to perform a maximum explosive pull, reaching as high as possible with one hand, without allowing any prior momentum or body sway. The recorded variable was the distance reached (cm) between the starting bar and the highest point touched. Two to three attempts were made on the dominant arm only, with 3–5 min between each, and the best valid attempt was recorded. Only attempts performed from a static position and with clear contact on the target bar were considered valid.

Intra-session reliability was examined using the three repeated trials collected for each main neuromuscular test. At baseline, ICC(3,1) values indicated excellent reliability for finger flexor maximum strength on the 20-mm hold for the right and left hands (0.971 and 0.962, respectively), good reliability for maximum isometric pull-up strength (0.764), and poor to moderate reliability for RFD-derived variables (range: 0.000–0.535). Accordingly, RFD outcomes were interpreted with caution due to their greater measurement variability.

### 2.6. Procedures

The study was conducted in three stages. In the first stage, participants completed a familiarization session prior to the initial tests, during which the evaluator explained and demonstrated each test, and participants performed submaximal practice trials. The initial assessment (pre-test) included body composition, pull-up repetition performance, maximum finger flexor strength and RFD with a 20-mm grip, maximum isometric strength during pull-ups, and RFD, as well as upper- and lower-body power tests.

The order of the tests was standardized as follows: body composition, vertical jump (CMJ), plyometric push-ups, Power Slap, pull-up repetition test, finger strength/RFD, and isometric pull-up strength/RFD. Prior to the performance tests, participants performed a standardized 10-min warm-up that included general mobility, low-intensity activation of the upper and lower body, and progressive submaximal practice attempts. Three attempts were made per test where applicable, and the best valid value was retained for analysis. Recovery intervals were standardized according to the demands of each test, ranging from 1 to 2 min for the jump and push-up tests, and from 3 to 5 min for the specific maximal climbing tests. For the Power Slap test, attempts were performed using only the dominant arm. Pre- and post-tests were conducted at the same climbing center, at the same time of day (±1 h), under similar environmental conditions, and using the same equipment and standardized protocols. To minimize external variability, participants were instructed to refrain from intense exercise for 48 h prior to each assessment, to maintain their usual sleep pattern for at least seven hours the night before, and to avoid altering their usual diet during the week preceding the measurements. No specific dietary intervention was implemented; however, participants were asked to maintain consistent nutritional habits during the assessment period. Dietary habits and supplement use were not formally monitored or recorded throughout the 10-week intervention period, which represents a limitation of the study.

To control training volume, the plyometric load was quantified by the number of reactive contacts per session, considering both explosive movements of the lower extremities (LE) and those of the upper extremities (UE). A reactive contact was defined as any explosive landing or foot strike in lower-body plyometric exercises, as well as any brief, reactive contact in explosive upper-body (UE) pulling exercises (campus board, plyometric pull-ups, and plyometric TRX). The volume per session was expressed as a range of contacts, since the effective load could vary slightly depending on the level of technical execution, individual selection of progressions, and the neuromuscular tolerance of each participant.

The second stage corresponded to the 10-week intervention (Table 2). During the adaptation phase (weeks 1–4), the volume ranged from 90 to 288 reactive contacts per session, at low to moderate intensities, prioritizing landing control, technical quality, and the progressive preparation of the musculotendinous structures. This initial progression was based on recommendations for the safe implementation of plyometric training in trained populations, where a prior adaptation phase is suggested before increasing intensity to optimize neuromuscular adaptations and reduce the risk of injury [16,17]. During the specific plyometric phase (weeks 5–6 and 8–10), the volume progressed to approximately 168–384 reactive contacts per session, at moderate to very high intensities, incorporating greater heights, increased eccentric demand, reduced contact time, and exercises highly transferable to bouldering technique. The seventh week was a deload week, aimed at reducing both volume and intensity, promoting neuromuscular recovery and consolidating previous adaptations, preserving the quality of the stimulus and reducing the risk of musculotendinous overload [16]. In the third stage, the final assessment (post-test) was conducted, replicating the initial protocols to ensure methodological consistency and control of experimental variability.

### 2.7. Ethical Considerations

The study was conducted in accordance with the regulations governing research involving human subjects and was reviewed and approved by the Scientific Ethics Committee of the University of Viña del Mar (CEC-UVM 32-25; date of approval: 8 July 2025). Furthermore, the ethical principles established in the Declaration of Helsinki for medical research involving human subjects [28] were observed. All participants signed an informed consent form detailing the procedures, potential risks and benefits, ensuring the confidentiality of the information and the right to withdraw voluntarily at any stage without consequences for their training program. This study was retrospectively registered in the ISRCTN registry (registration number: [ISRCTN10927935], date of registration: 22 June 2026).

### 2.8. Statistical Analysis

Data processing and statistical analysis were performed using Jamovi software (Version 2.4.11) (The Jamovi Project, Sydney, Australia). The assumptions of normality and homogeneity of variance were tested using the Shapiro–Wilk and Levene tests, respectively, given the small sample size and the potential influence of outliers on the model assumptions. When deviations from these assumptions were identified, the results were interpreted with caution, considering the exploratory nature of the study, the small sample size, and the absence of correction for multiple comparisons. A 2 × 2 mixed-design analysis of variance (group × time) was applied to examine the main effect of time, the main effect of group, and the group × time interaction. Since the time factor had only two levels, before and after, it was not necessary to assess the assumption of sphericity. To estimate the practical magnitude of the observed changes, effect sizes were calculated using the partial eta-squared statistic (η^2^p). Effect sizes estimated using η^2^p were interpreted according to Cohen’s [29] guidelines: small (η^2^p = 0.01), moderate (η^2^p = 0.06), and large (η^2^p = 0.14). Δ% was calculated as [(Post − Pre)/Pre] × 100 and used only as a descriptive indicator. To complement the inferential analysis and facilitate practical interpretation, mean changes (Δ = post − pre) were calculated for each variable, and between-group differences in change (Δ Difference = IG − GC) were compared using independent-samples Student’s *t*-tests with 95% confidence intervals (95% CI). The group × time interaction in the mixed ANOVA was considered the primary inferential criterion for evaluating differential changes between groups [30]. Comparisons between groups at baseline for Table 1 were performed using *t*-tests for independent samples, and effect sizes were reported as Hedges’ g. The level of statistical significance was set at *p* < 0.05. Given the exploratory nature of this study, no primary outcomes were specified prior to data collection; no correction for multiple comparisons was applied, and *p*-values are presented for descriptive purposes. Emphasis was placed on confidence intervals and effect sizes as indicators of practical relevance. All randomized participants completed the intervention and post-intervention assessments; therefore, the final analysis included all randomized participants with no missing outcome data and no need for imputation procedures. To address potential imbalances in baseline values, sensitivity analyses were conducted using ANCOVA for variables with notable differences between groups prior to the intervention, using the post-intervention score as the dependent variable, group as a fixed effect, and the baseline score as a covariate [31]. Intrasession reliability was estimated using the ICC(3,1), coefficient of variation (CV%), standard error of measurement (SEM), and the MDC95 based on the three repetitions performed for each test.

## 3. Results

Table 3 shows the results of the 2 × 2 mixed ANOVA. Significant group × time interactions were observed for pull-up performance (F = 5.26; *p* = 0.036; η^2^p = 0.248), with a moderate effect size; in the push-up power (F = 4.77; *p* = 0.044; η^2^p = 0.230) with a moderate effect; in the RFD 0–200 ms isometric pull-up (F = 8.10; *p* = 0.012; η^2^p = 0.336), with a large effect, and in the RFD 20–80% of isometric pull-up (F = 5.41; *p* = 0.034; η^2^p = 0.253) with a moderate-to-large effect. No significant group × time interaction was observed for the Power Slap (F = 0.72; *p* = 0.409; η^2^p = 0.043), finger flexor strength, finger RFD, maximum isometric pull-up strength, or CMJ. ANCOVA sensitivity analysis confirmed a significant group effect for isometric pull-up RFD 0–200 ms (F(1,15) = 8.69; *p* = 0.010; η^2^p = 0.367) and isometric pull-up RFD 20–80% (F(1,15)= 9.14; *p* = 0.009; η^2^p = 0.379), supporting the robustness of the main findings.

Table 4 presents the within-group changes and 95% confidence intervals. The intervention group showed the largest absolute change in pull-up performance (Δ = +6.00; 95% CI: 2.52, 9.48), isometric pull-ups at 200 ms RFD (Δ = +116.60; 95% CI: 30.95, 202.26), and isometric pull-up RFD at 20–80% of Fmax (Δ = +250.63; 95% CI: 44.24, 457.03). Percentage changes for RFD-derived variables are reported descriptively but should be interpreted cautiously because very low baseline values, floor effects, and lower intra-session reliability can inflate relative change estimates.

Table 5 presents between-group differences in change (Δ Difference = IG − CG), 95% confidence intervals, *p*-values, and effect sizes. Between-group differences favored the intervention group for pull-up performance, push-up power, isometric pull-up RFD 0–200 ms, and isometric pull-up RFD 20–80% of Fmax. The remaining variables showed no clear between-group differences.

## 4. Discussion

The main findings indicate that the 10-week plyometric program was associated with greater improvements than regular climbing training alone in certain neuromuscular outcomes assessed in the laboratory, specifically pull-up performance, power during plyometric push-ups, and isometric RFD during pull-ups. No clear differences were observed between the groups regarding finger flexor strength, finger RFD, maximum isometric strength during pull-ups, the Power Slap, or the CMJ. These findings should be interpreted as exploratory, especially given the small sample size, the absence of a primary outcome that specifies multiple comparisons, and the low reliability observed for several RFD-derived variables.

The greatest improvement was in isometric RFD during pull-ups in the intervention group, which may reflect the mechanical specificity of the training stimulus, which emphasized rapid force production in pulling movements. This interpretation is consistent with the general rationale of plyometric and ballistic training, which aims to improve the ability to generate force rapidly [16,17,19,20], and with previous evidence from climbing-related interventions that include plyometric, strength, or campus board stimuli [23,24]. However, no direct measures of neuromuscular activation, motor unit behavior, tendon stiffness, or muscle architecture were collected; therefore, possible explanations related to neural activation, motor unit firing rate, stretch-shortening cycle efficiency, or intermuscular coordination should be interpreted as hypotheses derived from the previous literature and not as mechanisms demonstrated in the present study [8,19,20].

The specificity of the improvement observed in isometric RFD during pull-ups can also be understood based on the mechanical characteristics of pulling movements. Pull-up tasks involve coordinated shoulder extension and adduction, elbow flexion, trunk stabilization, and the application of force against body mass or a fixed resistance [31,32,33,34,35,36]. In climbers, variations in pulling mechanics and grip configurations can influence grip demands and the biomechanics of the upper limbs [35,36]. Therefore, it is possible that the intervention was more closely aligned with the mechanical demands of isometric pull-up and plyometric push-up tests than with isolated results from finger flexors or the lower body. However, since the study did not include direct measures of climbing performance, these laboratory-observed improvements should not be interpreted as confirmed transfer to actual bouldering performance. Conversely, no clear differences were observed between the groups in terms of finger flexor strength or finger RFD. Finger-specific performance improvement may require targeted loading of the finger flexor through small- hold or grip-specific training protocols [24,37,38]. Conversely, repeated exposure to novel strength tests may produce learning or familiarization effects, particularly in maximal isometric tasks [39,40]. Previous studies suggest that specific finger training can improve finger strength and, in some cases, RFD-related outcomes in climbers [9,37]. In this study, the plyometric program did not include a specific load for the fingers; therefore, the absence of clear improvements related to the fingers can be interpreted in light of the principle of training specificity rather than as evidence that plyometric training is ineffective for all neuromuscular qualities relevant to climbing.

No clear difference was observed between the groups on the Power Slap test. Although the Power Slap test has been proposed as an assessment of climbing-specific power [27], the difference between the groups observed in the present study was modest and should not be interpreted as evidence of actual improved performance in bouldering. This test may capture a component of upper-body explosiveness, but it does not fully incorporate route reading, technical decision-making, center-of-mass control, movement efficiency, fatigue management, or sport-specific limitations. Previous climbing intervention studies have used climbing-specific or ecological outcomes, such as bouldering performance tests or lead climbing performance [24,41], highlighting the importance of including direct measures of climbing performance when transfer is the primary research question. Similarly, the absence of clear improvements in the CMJ may reflect the limited specificity of the lower-body intervention and its volume relative to the demands necessary to induce measurable adaptations in vertical jump. Although lower-body propulsion and intersegmental coordination are relevant to dynamic climbing movements [11,15], the present intervention focused predominantly on pulling and was not designed as a lower-body plyometric program. Therefore, the absence of clear changes in the CMJ is consistent with the characteristics of the stimulus and should not be interpreted as evidence that lower-body power is irrelevant for bouldering.

The large improvements in maximum isometric pull-up strength observed in both groups should also be interpreted with caution. Since the participants had no prior experience with this specific load-cell-based isometric pull-up testing protocol, the observed improvements may partly reflect familiarization, learning effects, and improved task performance rather than a physiological adaptation attributable to the intervention. This interpretation is consistent with evidence showing that repeated exposure to strength testing can produce substantial improvements through task practice and familiarization effects [39,40]. Consequently, future studies should incorporate specific familiarization sessions and reliability procedures across three sessions, particularly when using novel isometric testing protocols.

Finally, RFD results must be considered in light of measurement error. Intra-session reliability analysis showed excellent reliability for maximum finger flexor strength and good reliability for maximum strength and its metric in pull-ups, but low-to-moderate reliability for RFD-derived variables. Furthermore, the 80-Hz sampling rate of the portable load cell system limits the accuracy of force onset detection and RFD estimation in the initial phase; therefore, findings related to RFD should be interpreted in conjunction with absolute changes, confidence intervals, effect sizes, and measurement variability, rather than relying solely on percentage changes.

### 4.1. Practical Implications

From the perspective of pull-oriented plyometric training, this can be considered a complementary strategy to improve certain neuromuscular outcomes assessed in the laboratory, particularly pull-up performance, power during plyometric intervals, and isometric RFD during pull-ups. These findings are consistent with broader recommendations suggesting that resistance- and power-oriented training may contribute to the physical preparation of climbers when properly individualized and integrated into the training process [42]. However, the present study did not assess direct climbing performance outcomes such as completed boulder problems, attempts required, route-specific performance, or competition results. Therefore, these findings should not be interpreted as evidence that the intervention improves actual bouldering performance. Coaches should integrate plyometric stimuli according to the specific performance component they are targeting and should not assume a generalized transfer regarding finger strength, lower-body power, or ecological performance in climbing.

### 4.2. Limitations

Key limitations should be considered when interpreting the findings. First, the sample size was small, and no a priori sample size calculation was performed, which limited statistical power and generalizability. Second, no primary outcome was specified prior to data collection, and multiple outcomes were analyzed without correction for multiple comparisons, which increased the risk of false-positive findings. Third, several variables derived from RFD showed low intraclass reliability and high variability, particularly at baseline; therefore, RFD results should be interpreted with caution and with consideration of measurement error. Fourth, the 80-Hz sampling rate of the Tindeq Progressor^®^ limits the accuracy of force onset detection and RFD estimation during the initial phase.

Fifth, external training load was not objectively monitored via training logs, RPE per session, wearable devices, or quantification of external load, and some uncontrolled training exposure may have occurred. Sixth, no direct climbing performance outcomes, such as completed boulder problems, number of attempts, route-specific performance, or competition performance, were assessed. Therefore, the transfer to actual climbing performance cannot be confirmed. Other limitations include the sample consisting exclusively of men, the absence of formal monitoring of diet or supplements, potential familiarization effects on the maximum isometric pull-up test, the Power Slap assessment performed only with the dominant arm, and the single-blind design in which both participants and intervention supervisors were aware of group assignments.

Regarding recommendations for future studies, it would be useful to examine the strategic combination of maximum strength phases and power-oriented phases by progressively integrating higher volumes of specific reactive work on the Board campus in accordance with individualized progression criteria. Future research should also incorporate protocols for finger-specific maximum strength and rapid force development (RFD) to analyze potential synergistic effects between maximum finger strength and rapid force development in pulling actions. Another relevant line of research involves evaluating the direct transfer of improvements in rapid force development (RFD) obtained in the laboratory to ecological climbing indicators, such as completing boulders within a time limit, the number of attempts, route-specific efficiency, or competition performance. From a methodological perspective, future studies should increase sample sizes, consider multicenter designs, and include longer intervention periods to examine whether larger and more stable adaptations can be observed. Future research should also explore potential differences by sex, competitive level, and age, as well as integrate biomechanical, electromyographic, or musculotendinous measures to better understand the potential mechanisms underlying changes in neuromuscular performance in the laboratory.

## 5. Conclusions

Within the limitations of this preliminary exploratory study, a 10-week training program focused primarily on pull-oriented movements was associated with greater improvements in pull-up performance, plyometric push-up power, and isometric pull-ups RFD, compared to regular climbing training alone, in male boulder climbers with training levels ranging from trained to highly trained. No clear differences were observed between the groups in terms of finger flexor strength, finger RFD, maximum isometric strength on pull-ups, the Power Slap, or the CMJ. Given the exploratory design, the small sample size, the multiple comparisons, the low reliability of several RFD-derived variables, and the absence of direct results on bouldering performance, these findings should be interpreted with caution as preliminary evidence of improvements in movement-specific neuromuscular performance rather than as confirmation of improved bouldering performance.

## Figures and Tables

**Table 1:** Sample characteristics of the intervention and control groups.

Variable	Intervention Group Pre (n = 9)	Intervention Group Post (n = 9)	Control Group Pre (n = 9)	Control Group Post (n = 9)	Baseline p	Hedges’ g
Age (years)	29.67 ± 4.50	-	31.33 ± 5.63	-	0.500	−0.31
Height (cm)	173.33 ± 2.69	-	171.22 ± 7.89	-	0.465	0.34
Body mass (kg)	71.70 ± 9.40	71.84 ± 9.40	67.12 ± 12.18	67.01 ± 12.63	0.386	0.40
BMI (kg/m^2^)	23.87 ± 2.99	24.06 ± 2.79	22.73 ± 2.81	22.48 ± 2.92	0.417	0.37
Muscle mass (kg)	34.80 ± 3.47	34.71 ± 3.60	32.80 ± 6.58	33.38 ± 6.84	0.435	0.36
Body fat %	14.47 ± 5.15	14.83 ± 5.01	13.89 ± 5.65	12.50 ± 5.22	0.823	0.10

Note. Values are presented as mean ± SD. Age and height were recorded only at baseline. Baseline between-group comparisons were performed using an independent-samples *t*-test, and effect sizes are reported as Hedges’ g.

**Table 2:** Periodized plyometric training program across the 10-week intervention.

Week	Phase	Lower Body	Upper Body	Sets × Reps	Rest	Intensity	Reactive Contacts per Session
1	Adaptation	Depth Jumps, Lateral Bounds, Pogo Jumps.	Supported Campus, Plyometric Pull-ups, TRX Plyometric Rows	3 × 5	60–90	Low	90–180
2	Adaptation	Depth Jumps, Lateral Bounds, Squat Jump	Supported Campus, Plyometric Pull-ups, TRX Plyometric Row	3 × 6	60–90	Low	108–216
3	Adaptation	Depth Jumps, Lateral Bounds, Squat Jump	Single-leg step-ups, Plyometric pull-ups, TRX plyometric row	3 × 6	75	Medium	108–216
4	Adaptation	Depth Jumps, Lateral Bounds, Squat Jump	Single-leg step-ups, Plyometric pull-ups, TRX plyometric row	4 × 6	75	Moderate	144–288
5	Specific Plyometric Phase	Depth Jumps, Lateral Bounds, Squat Jump	Unassisted campus board sequence 1-5-7, Plyometric pull-ups, TRX plyometric row	4 × 7	90	High	168–336
6	Specific Plyometric Phase	Depth Jumps, Lateral Bounds, Squat Jump	Unassisted campus board sequence 1-4-5-5, Plyometric Pull-ups, TRX Plyometric Row	4 × 8	90	High	192–384
7	Deload	Depth Jumps, Lateral Bounds, Squat Jump Assisted campus board	One-arm pull-ups, Plyometric pull-ups, TRX plyometric row	3 × 5	75	Low	90–180
8	Specific Plyometric Phase	Depth Jumps, Lateral Bounds, Squat Jump	Double Campus Board Dynos 4-6-5-7-6, Pull-up Plyo, TRX Plyo Row	4 × 7	90	High	168–336
9	Specific Plyometric Phase	Depth Jumps, Lateral Bounds, Squat Jump	Unsupported parallel bars, double dynos 1-4-4-3-5-4, Plyometric pull-ups, TRX plyometric row	4 × 8	90	Very High	192–384
10	Specific Plyometric Phase	Depth Jumps, Lateral Bounds, Squat Jump	Unassisted Campus board sequence 1-5-3-1, Plyometric Pull-up, TRX Plyometric Row	4 × 8	90	Very High	192–384

Rest: inter-set recovery in seconds. Reactive contact per session includes all explosive ground contact (lower body) and reactive pull contacts (upper body). Campus sequences are expressed as run numbers. Intensity was defined as follows: Low = controlled landings, submaximal efforts; Moderate = increased eccentric demand, near-maximal effort; High = maximum reactive effort, reduced contact time; Very High = maximum intensity, complex sequences with minimal ground contact. Week 7 corresponds to a deload week aimed at reducing accumulated fatigue and consolidating prior adaptations.

**Table 3:** Results of the 2 × 2 mixed-design repeated-measures ANOVA for neuromuscular performance variables.

	Group			Time			Group × Time		
Variable	F	*p*	η^2^p	F	*p*	η^2^p	F	*p*	η^2^p
Pull-up	0.39	0.540	0.024	22.90	<0.001	0.589	5.26	0.036	0.248
Push-up Height (cm)	<0.01	0.969	0.000	0.78	0.391	0.046	1.47	0.243	0.084
Push-up power (W)	0.17	0.688	0.010	0.02	0.892	0.001	4.77	0.044	0.230
Power Slap (cm)	1.14	0.301	0.067	8.77	0.009	0.354	0.72	0.409	0.043
CMJ Height (cm)	1.12	0.307	0.065	14.94	0.001	0.483	2.66	0.122	0.143
CMJ Power (W)	1.93	0.184	0.107	7.20	0.016	0.310	2.93	0.106	0.155
Max Finger Flexor Strength, right hand, 20 mm (kg)	1.05	0.320	0.062	12.12	0.003	0.431	2.40	0.141	0.130
Max Finger Flexor Strength, left hand, 20 mm (kg)	1.08	0.315	0.063	9.92	0.006	0.383	0.12	0.733	0.007
Max Isometric Pull-up Strength (kg)	6.01	0.026	0.273	73.38	<0.001	0.821	2.41	0.140	0.131
RFD 0–200 ms Right Fingers (kg/s)	1.04	0.323	0.061	8.18	0.011	0.338	0.05	0.828	0.003
RFD 0–200 ms Left Fingers (kg/s)	8.07	0.012	0.335	3.81	0.069	0.192	0.15	0.700	0.010
RFD 20–80% Right Fingers (kg/s)	1.73	0.207	0.098	0.95	0.345	0.056	1.94	0.182	0.108
RFD 20–80% Left Fingers (kg/s)	5.94	0.027	0.271	0.84	0.373	0.050	1.42	0.252	0.081
RFD 0–200 ms Isometric Pull-up (kg/s)	8.93	0.009	0.358	10.95	0.004	0.406	8.10	0.012	0.336
RFD 20–80% Isometric Pull-up (kg/s)	9.69	0.007	0.377	4.52	0.013	0.330	5.41	0.034	0.253

Note. The results are presented as F-statistics, *p*-values, and effect sizes (η^2^p) from the 2 × 2 mixed-design repeated-measures ANOVA. All repeated-measures ANOVA effects used df = 1.16. For ANCOVA sensitivity analyses, df = 1.15. CMJ: countermovement jump; RFD: rate of force development.

**Table 4:** Pre- and post-intervention results, percentage changes (Δ%), and mean changes with 95% confidence intervals (95% CI) for neuromuscular performance variables in the intervention group (IG) and control group (CG).

Variable	Group	Pre (Mean ± SD)	Post (Mean ± SD)	Δ%	Δ (95%CI)
Pull-up (reps)	IG	17.11 ± 3.33	23.11 ± 6.25	+35.06%	+6.00 (2.52, 9.48)
	CG	17.33 ± 6.54	19.44 ± 7.47	+12.18%	+2.11 (0.33, 3.89)
Push-up Height (cm)	IG	10.16 ± 4.13	10.46 ± 1.96	+2.96%	+0.30 (−2.66, 3.26)
	CG	11.20 ± 4.65	9.30 ± 3.00	−16.96%	−1.90 (−4.85, 1.06)
Push-up Power (W)	IG	915.83 ± 225.62	997.45 ± 144.18	+8.91%	+81.62 (−38.00, 201.24)
	CG	963.71 ± 301.37	871.08 ± 177.41	−9.61%	−92.63 (−232.52, 47.26)
Power Slap (cm)	IG	81.11 ± 8.72	87.22 ± 5.12	+7.53%	+6.11 (0.08, 12.14)
	CG	76.11 ± 15.89	79.50 ± 18.14	+4.45%	+3.39 (−0.89, 7.67)
CMJ Height (cm)	IG	34.13 ± 5.75	37.74 ± 4.02	+10.56%	+3.61 (1.73, 5.48)
	CG	32.71 ± 5.39	34.18 ± 5.42	+4.48%	+1.47 (−0.91, 3.84)
CMJ Power (W)	IG	869.36 ± 138.35	948.94 ± 107.42	+9.15%	+79.59 (1.60, 157.57)
	CG	822.36 ± 131.05	839.97 ± 122.08	+2.14%	+17.61 (−12.32, 47.54)
Max Finger Flexor Strength, right hand, 20 mm (kg)	IG	56.13 ± 6.41	60.18 ± 6.21	+7.21%	+4.05 (1.27, 6.82)
	CG	52.85 ± 12.34	54.41 ± 11.35	+2.94%	+1.56 (−0.91, 4.02)
Max Finger Flexor Strength, left hand, 20 mm (kg)	IG	55.51 ± 6.74	57.95 ± 7.06	+4.40%	+2.44 (1.01, 3.87)
	CG	51.27 ± 10.97	53.23 ± 11.34	+3.81%	+1.96 (−0.93, 4.84)
RFD 200 ms Right Fingers (kg/s)	IG	53.82 ± 83.94	126.17 ± 96.89	+134.43%	+72.35 (−12.61, 157.32)
	CG	30.14 ± 51.81	92.13 ± 72.11	+205.61%	+61.98 (−5.15, 129.11)
RFD 200 ms Left Fingers (kg/s)	IG	77.16 ± 97.63	151.56 ± 120.67	+96.43%	+74.40 (−58.22, 207.02)
	CG	17.97 ± 30.68	67.50 ± 67.29	+275.65%	+49.53 (−12.33, 111.39)
RFD 20–80% Right Fingers (kg/s)	IG	154.33 ± 92.16	215.40 ± 61.42	+39.57%	+61.07 (−44.77, 166.91)
	CG	143.88 ± 126.84	133.05 ± 77.51	−7.52%	−10.82 (−65.10, 43.45)
RFD 20–80 Left Fingers (kg/s)	IG	168.97 ± 111.18	221.46 ± 75.21	+31.07%	+52.49 (−28.51, 133.50)
	CG	105.89 ± 118.66	99.10 ± 71.70	−6.41%	−6.78 (−88.28, 74.71)
RFD 200 ms Pull-up (kg/s)	IG	24.41 ± 58.21	141.02 ± 115.09	+477.65%	+116.60 (30.95, 202.26)
	CG	5.99 ± 11.46	14.74 ± 15.58	+146.29%	+8.76 (−8.41, 25.92)
RFD 20–80% Pull-up (kg/s)	IG	107.29 ± 120.10	357.92 ± 233.28	+233.7%	+250.63 (44.24, 457.03)
	CG	81.78 ± 90.03	70.64 ± 159.75	−13.63%	−11.15 (−168.68, 146.39)
Max Isometric Pull-up Strength (Kg)	IG	32.90 ± 5.20	69.57 ± 9.94	+111.45%	+36.67 (26.55, 46.79)
	CG	28.26 ± 4.98	53.68 ± 20.04	+89.97%	+25.42 (12.12, 38.73)

Values are presented as mean ± standard deviation (SD); Δ: mean change (post − pre); (Δ%): Percentage changes [(Post − Pre)/Pre] × 100. IG: intervention group; CG: control group; CMJ: countermovement jump; RFD: rate of force development. A confidence interval for Δ that does not include zero indicates a statistically significant within-group change.

**Table 5:** Comparison of changes (Δ) in neuromuscular performance variables, including Δ for the difference (IG–CG), 95% confidence intervals (95% CI), *p*-values, and effect sizes (η^2^p).

Variable	Δ IG (95% CI)	Δ CG (95% CI)	Δ Difference (95% CI)	*p*	η^2^p
Pull-up (reps)	+6.00 (2.52, 9.48)	+2.11 (0.33, 3.89)	+3.89 (0.30, 7.48)	0.036	0.248
Push-up Height (cm)	+0.30 (−2.66, 3.26)	−1.90 (−4.85, 1.06)	+2.20 (−1.64, +6.04)	0.243	-
Push-up Power (W)	+81.62 (−38.00, 201.24)	−92.63 (−232.52, 47.26)	+174.25 (5.05, 343.46)	0.044	0.128
Power Slap (cm)	+6.11 (−4.08, 9.52)	+3.39 (−7.67, 0.89)	+2.72 (−4.08, 9.52)	0.409	-
CMJ Height (cm)	+3.61 (1.73, 5.48)	+1.47 (−0.91, 3.84)	+2.14 (−0.64, 4.92)	0.122	-
CMJ Power (W)	+79.59 (+1.60, 157.57)	+17.61 (−12.32, 47.54)	+61.98 (−14.82, 138.77)	0.106	-
Max Finger Flexor Strength, right hand, 20 mm (kg)	+4.05 (+1.27, 6.82)	+1.56 (−0.91, 4.02)	+2.49(−0.88, 5.86)	0.141	-
Max Finger Flexor Strength, left hand, 20 mm (kg)	+ 2.44 (+1.01, 3.87)	+ 1.96 (−0.93, 4.84)	+0.48 (−2.47, 3.44)	0.733	-
RFD 0–200 ms Right Fingers (kg/s)	+72.35 (−12.61, 157.32)	+61.98 (−5.15, +129.11)	+10.37 (−89.17, 109.91)	0.828	-
RFD 0–200 ms Left Fingers (kg/s)	+74.40 (−58.22, 207.02)	+49.53 (−12.33, 111.39)	+24.87 (−109.66, 159.40)	0.700	-
RFD 20–80% Right Fingers (kg/s)	+61.07 (−44.77, +166.91)	−10.82 (−65.10, 43.45)	+71.89 (−37.45, 181.24)	0.182	-
RFD 20–80% Left Fingers (kg/s)	+52.49 (−28.51, 133.50)	−6.78 (−88.28, 74.71)	+59.28 (−46.35, 164.91)	0.252	-
RFD 0–200 ms Pull-up (kg/s)	+116.60 (27.54, 188.16)	+8.76 (−8.41, +25.92)	+107.85 (27.54, 188.16)	0.012	0.336
RFD 20–80% Pull-up (kg/s)	+250.63 (23.19, 500.47)	−11.15 (−168.68, 146.39)	+261.78 (23.09, 500.47)	0.034	0.253
Max Isometric Pull-up Strength (Kg)	+36.67 (26.55, 46.79)	+25.42 (12.12, 38.73)	+11.24 (−4.12, 26.61)	0.140	-

Values are presented as mean changes (Δ) with 95% confidence intervals (95% CI) for the intervention group (IG) and control group (CG), and between-group differences in change (Δ difference), along with *p*-values and effect sizes (η^2^p). A *p*-value of <0.05 was considered statistically significant. For the Δ Difference column, a confidence interval that does not include the value zero indicates a statistically significant difference in changes between groups.

## Data Availability

The data presented in this study are openly available in OSF at: https://doi.org/10.17605/OSF.IO/NWFMB.

## Abbreviations

The following abbreviations are used in this manuscript:

Abbreviation	Definition
ANOVA	Analysis of variance
CG	Control group
CI	Confidence interval
CMJ	Countermovement jump
ICC	Intraclass correlation coefficient
IG	Intervention group
IRCRA	International Rock Climbing Research Association
RFD	Rate of force development
SD	Standard deviation
η^2^p	Partial eta-squared
